# The epidemiology of tuberculosis infection and the Human Development Index (HDI) in Iran between 1990–2021: secondary analysis of the global burden of disease

**DOI:** 10.1186/s12879-026-13148-2

**Published:** 2026-04-11

**Authors:** Afsaneh Kaffash, Saeideh Sadat Shobeiri, Elham Pouraslan, Asghar Kazemzadeh, Fatemeh Doost Mohammadi, Nima Ghazal, Mohamad Amini, Habib Ashena, Zaher Khazaei

**Affiliations:** 1 Clinical Research Development Unit, Vasei Hospital, Sabzevar University of Medical Sciences, Sabzevar, Iran; 2 Department of Obstetrics & Gynecology, Faculty of Medicine, Sabzevar University of Medical Sciences, Sabzevar, Iran; 3 Clinical Research Development Unit, Shahid Bahonar Hospital, Kerman University of Medical Sciences, Kerman, Iran; 4 Center for Healthcare Data Modeling, Departments of Biostatistics and Epidemiology, School of Public Health, Shahid Sadoughi University of Medical Sciences, Yazd, Iran; 5 Student Research Committee, School of Public Health, Shahid Sadougi University of Medical Sciences of Yazd, Yazd, Iran; 6 Non-Communicable Diseases Research Center, Sabzevar University of Medical Sciences, Sabzevar, Iran

**Keywords:** Tuberculosis, Multidrug-resistant, Extensively drug-resistant, Latent infection, Human development index

## Abstract

**Objective:**

This ecological study examines epidemiological trends in tuberculosis, multidrug-resistant TB, extensively drug-resistant TB, and latent TB infection across Iran and its provinces from 1990 to 2021, and explores their association with the Human Development Index.

**Methods:**

Data from the Global Burden of Disease 2021 study, were used to assess the incidence, mortality, and prevalence of tuberculosis, multidrug-resistant tuberculosis, extensively drug-resistant tuberculosis, and latent tuberculosis infection at both national and provincial levels in Iran. The association between tuberculosis indicators and the Human Development Index was evaluated using bivariate correlation analysis, with *P* < 0.05 considered statistically significant. Geographic maps illustrating the spatial distribution of the disease across provinces were generated using Arc-GIS 10.8.2 software. All statistical analyses were performed using Stata version 12 (Stata Corp, College Station, TX, USA).

**Results:**

The age-standardized incidence rate (ASIR) of tuberculosis decreased from 22.60 cases per 100,000 population (95% CI: 20.09–25.41) in 1990 to 13.51 cases (95% CI: 11.98–15.15) in 2021. The average annual percent change was: − 0.40 (95% CI: − 0.43 to − 0.37). In 2021, the age-standardized mortality rate was 1.09 cases per 100,000 (95% CI: 0.92–1.30), with an average annual percent change: − 0.73; (95% CI: − 0.80 to − 0.57). Sistan and Baluchistan (32.21 per 100,000) and Golestan (24.46 per 100,000) reported the highest incidence rates, whereas Chahar Mahaal and Bakhtiari (3.08 per 100,000) and Isfahan (6.71 per 100,000) reported the lowest. A significant inverse correlation was observed between the Human Development Index and tuberculosis indicators (*P* < 0.05). The prevalence of multidrug-resistant tuberculosis was 1.7%, and the prevalence of latent tuberculosis infection was 25,027 cases per 100,000 population.

**Conclusion:**

Substantial progress has been made in reducing the tuberculosis burden in Iran. Nevertheless, marked regional disparities and persistent challenges related to multidrug-resistant tuberculosis and latent TB infection highlight the need for targeted interventions, including enhanced screening, improved diagnostic capacity, and expanded preventive treatment strategies.

## Introduction

Tuberculosis (TB) is a preventable and largely curable disease. In 2023, TB reemerged as the leading cause of death from a single infectious agent worldwide, surpassing coronavirus disease 2019 (COVID‑19) after three years and accounting for nearly twice as many deaths as human immunodeficiency virus/acquired immunodeficiency syndrome (HIV/AIDS) [1]. Each year, TB affects more than 10 million people worldwide, and since 2021, the number of new cases has been on the rise [2].

Intensified action is required to achieve the global objective of ending the TB epidemic by 2030, a commitment formally endorsed by all United Nations member states [3, 4]. The advent and broad implementation of effective anti‑tuberculosis antimicrobials in the mid‑20th century resulted in a significant reduction in TB‑related mortality [3]. However, despite substantial progress in chemotherapy, tuberculosis re-emerged as the leading infectious cause of death seven decades later. In 2016, TB was responsible for 1.3 million deaths among individuals living with HIV, surpassing overall HIV‑related mortality. Additionally, TB accounted for 374,000 HIV‑associated deaths, establishing it as the primary cause of mortality in HIV‑positive populations. Globally, TB continues to disproportionately affect impoverished communities, and, many patients experience long‑term sequelae after treatment that markedly impair quality of life [5, 6].

Despite the availability of effective therapies for TB, global control efforts are hindered by the emergence of multidrug‑resistant tuberculosis (MDR‑TB) and extensively drug‑resistant tuberculosis (XDR‑TB) strains. MDR/XDR‑TB poses formidable challenges, including low detection rates, high treatment failure, and substantially increased mortality [7, 8]. The increase in MDR/XDR-TB cases is largely attributed to limitations in diagnostic tests for pathogen drug susceptibility, which enable continued transmission through close contact. Inadequate or inconsistent drug regimens further contribute to the development of additional drug resistance [9]. Individuals with close contact to MDR/XDR‑TB cases face a at markedly increased risk of infection [10, 11]. Infectious aerosol particles must be between 1 and 5 micrometres in diameter to reach the alveoli and initiate TB infection [9, 12]. The risk of progression to active TB is highest within the first 12 to 18 months after initial infection, although reactivation may occur decades later [13, 14].

Conditions that compromise innate or adaptive immunity substantially increase the risk of disease progression in individuals with latent tuberculosis infection (LTBI) [13, 15]. Approximately one‑quarter of the global population, or about 2 billion individuals, are estimated to harbour latent tuberculosis infection (LTBI), carrying the bacteria without progressing to active disease [13]. The prevalence of LTBI varies markedly across different regions and populations [16]. Variations in TB incidence and mortality are influenced by factors such as impaired immunity, malnutrition, comorbidities, and a range of socioeconomic determinants, including education, socioeconomic status, societal roles, and overall societal development [17].

The Human Development Index (HDI), which is calculated using life expectancy at birth, education, and per capita income, exhibits both direct and inverse relationships with disease incidence, prevalence, and mortality across populations [18]. For example, cancer incidence frequently increases with higher HDI, while mortality from these diseases generally decreases as HDI rises [19–21]. The present study’s findings are consistent with international research, highlighting a correlation between tuberculosis (TB) and the HDI [10, 17, 22, 23]. Although significant global efforts have been made to control TB, important gaps persist in understanding its epidemiological patterns at the subnational level, especially in middle-income countries such as Iran. In these settings, regional disparities and socioeconomic conditions significantly affect disease dynamics [22, 23]. Most prior research has concentrated on national tuberculosis trends or international comparisons, often neglecting the complex interactions between disease burden—including multidrug-resistant TB (MDR-TB), extensively drug-resistant TB (XDR-TB), and latent TB infection (LTBI)—and key indicators such as the HDI.

This study address critical knowledge gaps by using data from the Global Burden of Disease (GBD) 2021 to evaluate trends in TB incidence, prevalence, and mortality in Iran from 1990 to 2021. The analysis highlights the association between these trends and the HDI, and examines subnational disparities across provinces. Spatial analysis performed with ArcGIS provides a comprehensive overview of TB epidemiology in Iran, providing insights to guide targeted health interventions and inform policy decisions aligned with the global objectives of ending the TB epidemic by 2030.

## Methods

### Data source

The Institute for Health Metrics and Evaluation (IHME) provides annual updates of the GBD study, which examines temporal and geographical trends from 1990 onward to guide health policy and resource allocation. The GBD 2021 study estimated incidence, prevalence, mortality, and disability‑adjusted life years (DALYs) for 354 diseases and injuries, along with 3,484 sequelae and disabling outcomes, stratified by age, sex, year, and location [24, 25].

This ecological study investigated the distribution of incidence, mortality, MDR‑TB, and LTBI prevalence in Iran, as well as their association with the HDI. Data were obtained from publicly available sources at http://ghdx.healthdata.org/gbd-results-tool. The extracted dataset included estimates of incidence, mortality, and prevalence across all age and sex groups, each with corresponding 95% confidence intervals (95% CI). The Average Annual Percent Change (AAPC) was also calculated for selected indicators.

### Epidemiological

TB incidence refers to the number of new cases per 100,000 population, whereas while prevalence encompasses both new and existing cases. Mortality denotes the number of deaths per 100,000 population. The Global Burden of Disease (GBD) study compiles these metrics using multiple data sources.

HDI data were obtained from the United Nations Development Programme (UNDP).The HDI is a composite measure of national achievements in health, education, and living standards, and the United Nations publishes annual HDI rankings for its member states [18]. The AAPC quantifies trends over a specified interval by providing a single value that reflects the average annual percent change, irrespective of trend shifts. It is derived as a weighted average of the Annual Percent Changes (APCs) estimated using the join point regression model.

$$\:AAPC=\{\mathrm{exp}\left(\frac{\sum\:wibi}{\sum\:wi}\right)-1\}\times\:100$$

The HDI was calculated as the geometric mean of normalized indices across three dimensions: life expectancy at birth, education (measured by mean years of schooling among adults aged 25 years and older and expected years of schooling for children), and gross national income per capita, which was log‑transformed to account for diminishing returns [18, 19]. Spatial patterns of TB incidence, prevalence, and mortality across Iran’s provinces were analyzed using ArcGIS version 10.8.2. The software was applied to visualize high‑ and low‑risk areas, thereby enhancing understanding of the disease’s geographic distribution and intensity [28]. Bivariate correlation analysis was conducted to assess the relationship between TB and the HDI, with *P* < 0.05 set as the threshold for statistical significance.All data analyses were performed using Stata version 14 (Stata Corp, College Station, TX, USA) [20].

## Results

### Trend analysis

The findings indicate a sustained decline in TB incidence, mortality, and prevalence in Iran between 1990 and 2021. In 2021, the incidence rate for both sexes was 13.51 per 100,000 population [95% confidence interval 95% CI:)11.98–15.15)], the mortality rate was 1.09 per 100,000 [95% CI:)0.92–1.34(], and the prevalence of LTBI was 25,046 per 100,000 [95% CI:)22,373–28,088(]. Incidence and mortality rates in Iran remain significantly lower than the global average, reflecting a comparatively favorable epidemiological profile.

The AAPC from 1990 to 2021 was − 0.40 for TB incidence [95% confidence interval (CI: −0.43, − 0.37(], − 0.73 for mortality [95% CI:) − 0.80, − 0.57(], and − 0.11 for LTBI [95% CI:) − 0.14, 0.08(], confirming a consistent downward trend during the study period. The GBD report shows that the incidence rate declined from 22.60 per 100,000 [95% CI:)20.09, 25.41(] in 1990 to 17.52 [95% CI:)15.61, 19.56(] in 2000, 15.65 [95% CI:)13.85, 17.43(] in 2010, and 13.51 [95% CI:)11.98, 15.15(] per 100,000 in 2021. TB mortality and prevalence rates also declined steadily throughout this period.

**Fig. 1:**
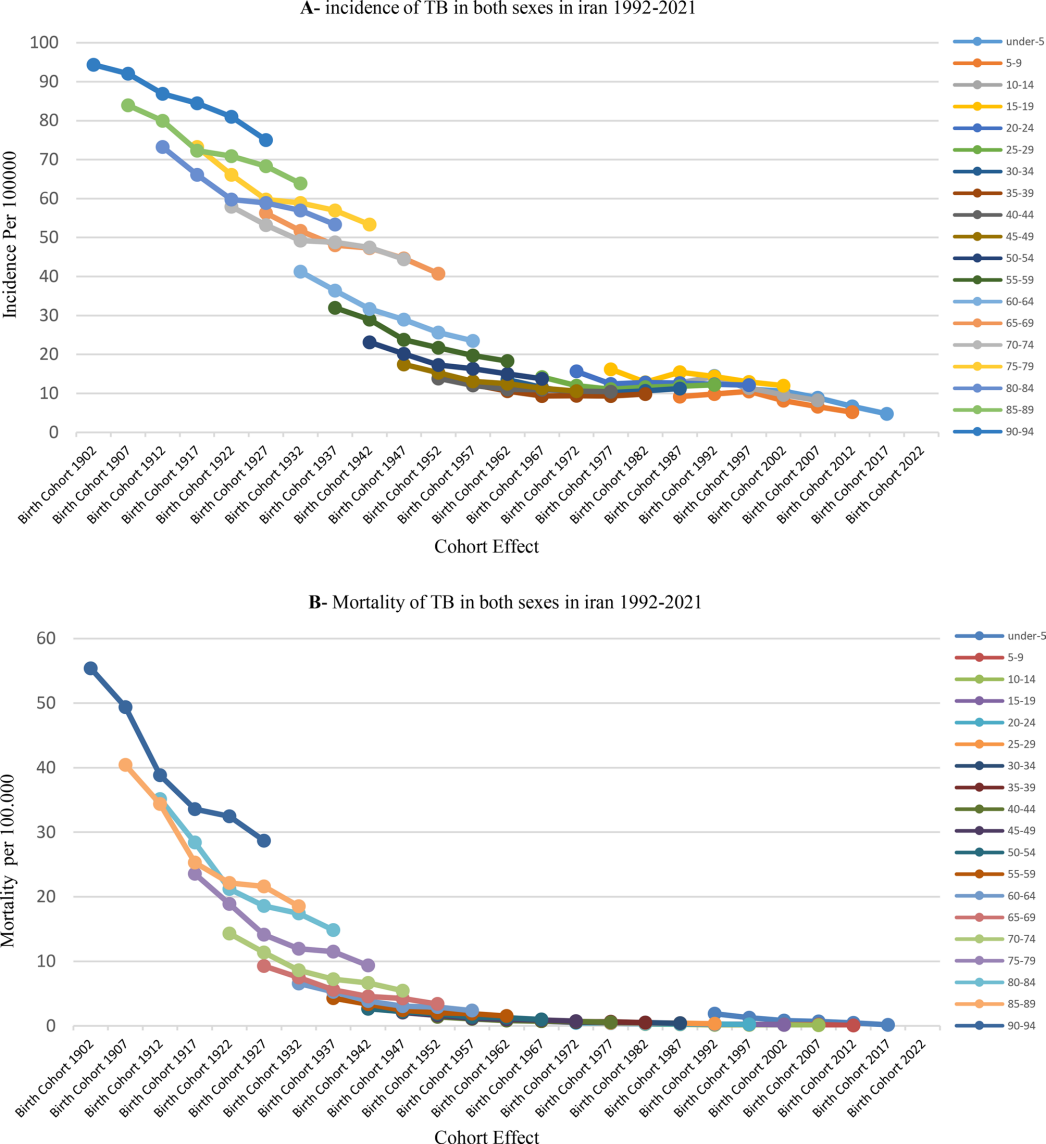
Incidence **(A)** and mortality **(B)** from TB in the Iranian population during the years 1992–2021

Figure 1 shows that TB incidence increases with age (Graph A), while more recent birth cohorts exhibit lower incidence compared to older cohorts. Similarly, Graph B demonstrates that TB mortality rises with age, yet newer cohorts experience lower mortality rates than earlier generations. The decline in both incidence and mortality among recent cohorts is likely attributable to effective screening, timely diagnosis and treatment, and universal access to anti‑tuberculosis medications in Iran. Age effects reflect biological and social changes associated with aging that influence disease risk. Period effects represent broad environmental, social, or medical factors affecting all individuals within a specific timeframe, regardless of age. Cohort effects capture shared exposures or experiences, often tied to birth year, that shape health outcomes later in life.

**Table 1:** Tuberculosis; cases of drug-sensitive, MDR, and XDR incidence in Iran, 2021

Region	Drug-Sensitive			Multidrug-Resistant Without Extensively Drug-Resistant			Extensively Drug-Resistant			Total tuberculosis cases		
	Male	Female	both	Male	Female	both	Male	Female	both	Male	Female	both
Alborz	12.44 (10.57, 14.25)	12.68 (10.85, 14.64)	12.49 (10.68, 14.34)	0.28 (0.02, 1.17)	0.30 (0.03, 1.26)	0.29 (0.03, 1.20)	0.01 (0.00, 0.04)	0.01 (0.00, 0.05)	0.01 (0.00, 0.05)	12.73 (11.08, 14.62)	12.99 (11.34, 14.79)	12.79 (11.20, 14.59)
Ardebil	9.12 (7.80, 10.57)	8.12 (6.97, 9.30)	8.60 (7.41, 9.81)	0.18 (0.01, 0.77)	0.16 (0.01, 0.67)	0.17 (0.01, 0.71)	0.01 (0.00, 0.03)	0.01 (0.00, 0.02)	0.01 (0.00, 0.03)	9.31 (8.06, 10.70)	8.29 (7.30, 9.38)	8.78 (7.69, 9.93)
Bushehr	11.92 (10.29, 13.67)	8.52 (7.38, 9.76)	10.27 (8.89, 11.70)	0.26 (0.02, 1.06)	0.18 (0.02, 0.74)	0.22 (0.02, 0.90)	0.01 (0.00, 0.04)	0.01 (0.00, 0.03)	0.01 (0.00, 0.03)	12.19 (10.56, 13.89)	8.71 (7.62, 9.89)	10.49 (9.21, 11.89)
Chahar Mahaal and Bakhtiari	3.36 (2.81, 3.98)	2.67 (2.24, 3.11)	3.01 (2.51, 3.51)	0.08 (0.01, 0.34)	0.07 (0.00, 0.27)	0.07 (0.01, 0.31)	0.00 (0.00, 0.01)	0.00 (0.00, 0.01)	0.00 (0.00, 0.01)	3.45 (2.92, 4.05)	2.74 (2.34, 3.16)	3.08 (2.66, 3.53)
East Azarbayejan	9.26 (7.48, 11.27)	8.21 (6.75, 9.83)	8.74 (7.24, 10.52)	0.18 (0.01, 0.78)	0.15 (0.01, 0.70)	0.17 (0.01, 0.73)	0.01 (0.00, 0.03)	0.01 (0.00, 0.03)	0.01 (0.00, 0.03)	9.44 (7.60, 11.52)	8.37 (6.93, 10.05)	8.91 (7.31, 10.73)
Fars	9.91 (8.95, 11.08)	7.17 (6.33, 8.15)	8.56 (7.71, 9.56)	0.19 (0.02, 0.71)	0.14 (0.01, 0.53)	0.16 (0.02, 0.63)	0.01 (0.00, 0.03)	0.00 (0.00, 0.02)	0.01 (0.00, 0.02)	10.10 (9.11, 11.26)	7.31 (6.46, 8.29)	8.73 (7.91, 9.70)
Gilan	11.52 (10.01, 13.19)	7.07 (6.10, 8.08)	9.31 (8.07, 10.55)	0.23 (0.02, 0.78)	0.14 (0.01, 0.47)	0.19 (0.02, 0.62)	0.01 (0.00, 0.03)	0.01 (0.00, 0.02)	0.01 (0.00, 0.02)	11.76 (10.25, 13.51)	7.22 (6.30, 8.24)	9.50 (8.34, 10.77)
Golestan	28.00 (24.31, 32.19)	20.13 (17.44, 22.93)	23.98 (21.02, 27.44)	0.54 (0.04, 2.20)	0.38 (0.03, 1.59)	0.46 (0.04, 1.89)	0.02 (0.00, 0.09)	0.01 (0.00, 0.06)	0.02 (0.00, 0.07)	28.56 (25.10, 32.46)	20.53 (18.06, 23.41)	24.46 (21.72, 27.65)
Hamadan	8.00 (6.88, 9.23)	6.72 (5.82, 7.75)	7.37 (6.43, 8.48)	0.15 (0.01, 0.62)	0.13 (0.01, 0.52)	0.14 (0.01, 0.56)	0.01 (0.00, 0.02)	0.00 (0.00, 0.02)	0.01 (0.00, 0.02)	8.16 (7.06, 9.41)	6.85 (5.98, 7.82)	7.52 (6.59, 8.54)
Hormozgan	19.18 (16.56, 21.89)	10.53 (9.23, 11.95)	14.91 (13.17, 16.87)	0.36 (0.03, 1.40)	0.19 (0.02, 0.78)	0.28 (0.03, 1.09)	0.01 (0.00, 0.06)	0.01 (0.00, 0.03)	0.01 (0.00, 0.04)	19.55 (17.10, 22.26)	10.73 (9.42, 12.14)	15.20 (13.39, 17.06)
Ilam	8.05 (6.96, 9.34)	7.00 (6.04, 8.03)	7.52 (6.51, 8.66)	0.18 (0.02, 0.66)	0.15 (0.01, 0.55)	0.16 (0.02, 0.59)	0.01 (0.00, 0.03)	0.01 (0.00, 0.02)	0.01 (0.00, 0.02)	8.24 (7.20, 9.49)	7.16 (6.26, 8.19)	7.68 (6.73, 8.84)
Isfahan	6.73 (5.70, 7.83)	6.39 (5.49, 7.41)	6.57 (5.60, 7.53)	0.14 (0.01, 0.57)	0.13 (0.01, 0.53)	0.14 (0.01, 0.55)	0.01 (0.00, 0.02)	0.00 (0.00, 0.02)	0.00 (0.00, 0.02)	6.88 (5.96, 7.95)	6.53 (5.68, 7.44)	6.71 (5.87, 7.66)
Kerman	12.48 (10.82, 14.22)	11.50 (9.99, 13.19)	12.02 (10.63, 13.57)	0.23 (0.02, 0.89)	0.21 (0.02, 0.82)	0.22 (0.02, 0.86)	0.01 (0.00, 0.04)	0.01 (0.00, 0.03)	0.01 (0.00, 0.03)	12.71 (11.06, 14.40)	11.71 (10.26, 13.29)	12.25 (10.82, 13.76)
Kermanshah	16.20 (14.72, 17.75)	13.35 (12.01, 14.78)	14.79 (13.52, 16.00)	0.28 (0.03, 1.16)	0.24 (0.02, 0.98)	0.26 (0.03, 1.07)	0.01 (0.00, 0.04)	0.01 (0.00, 0.03)	0.01 (0.00, 0.04)	16.50 (15.16, 17.93)	13.60 (12.37, 15.15)	15.06 (13.87, 16.30)
Khorasan-e-Razavi	17.73 (15.41, 20.05)	16.30 (14.16, 18.51)	17.01 (14.91, 18.99)	0.31 (0.03, 1.20)	0.28 (0.03, 1.07)	0.30 (0.03, 1.12)	0.01 (0.00, 0.05)	0.01 (0.00, 0.04)	0.01 (0.00, 0.04)	18.05 (15.91, 20.40)	16.59 (14.61, 18.65)	17.32 (15.31, 19.34)
Khuzestan	19.33 (16.53, 22.41)	13.50 (11.72, 15.46)	16.45 (14.32, 18.77)	0.37 (0.04, 1.53)	0.26 (0.03, 1.07)	0.31 (0.03, 1.29)	0.01 (0.00, 0.06)	0.01 (0.00, 0.04)	0.01 (0.00, 0.05)	19.71 (17.15, 22.69)	13.77 (12.10, 15.67)	16.77 (14.85, 19.04)
Kohgiluyeh and Boyer-Ahmad	7.45 (6.40, 8.59)	8.04 (6.98, 9.35)	7.74 (6.79, 8.94)	0.16 (0.02, 0.68)	0.18 (0.02, 0.72)	0.17 (0.02, 0.70)	0.01 (0.00, 0.02)	0.01 (0.00, 0.03)	0.01 (0.00, 0.02)	7.62 (6.60, 8.72)	8.22 (7.20, 9.41)	7.92 (6.96, 9.08)
Kurdistan	8.29 (7.11, 9.44)	8.68 (7.48, 9.91)	8.50 (7.42, 9.70)	0.17 (0.02, 0.65)	0.18 (0.02, 0.69)	0.17 (0.02, 0.67)	0.01 (0.00, 0.02)	0.01 (0.00, 0.02)	0.01 (0.00, 0.02)	8.46 (7.41, 9.64)	8.86 (7.82, 10.02)	8.68 (7.59, 9.78)
Lorestan	14.94 (12.93, 17.32)	9.54 (8.28, 11.00)	12.13 (10.55, 14.02)	0.32 (0.03, 1.29)	0.20 (0.02, 0.81)	0.26 (0.02, 1.03)	0.01 (0.00, 0.05)	0.01 (0.00, 0.03)	0.01 (0.00, 0.04)	15.28 (13.34, 17.60)	9.75 (8.47, 11.17)	12.40 (10.91, 14.19)
Markazi	11.36 (9.75, 13.09)	10.94 (9.42, 12.48)	11.17 (9.64, 12.73)	0.23 (0.02, 0.92)	0.22 (0.02, 0.90)	0.23 (0.02, 0.91)	0.01 (0.00, 0.03)	0.01 (0.00, 0.03)	0.01 (0.00, 0.03)	11.60 (10.10, 13.26)	11.17 (9.86, 12.63)	11.41 (10.06, 12.84)
Mazandaran	9.96 (8.54, 11.38)	6.98 (5.96, 8.06)	8.47 (7.30, 9.65)	0.21 (0.02, 0.82)	0.15 (0.01, 0.58)	0.18 (0.02, 0.70)	0.01 (0.00, 0.03)	0.01 (0.00, 0.02)	0.01 (0.00, 0.03)	10.18 (8.75, 11.56)	7.13 (6.22, 8.22)	8.66 (7.55, 9.81)
North Khorasan	10.00 (8.55, 11.54)	8.82 (7.67, 10.09)	9.42 (8.17, 10.70)	0.20 (0.02, 0.87)	0.17 (0.01, 0.79)	0.19 (0.01, 0.84)	0.01 (0.00, 0.03)	0.01 (0.00, 0.03)	0.01 (0.00, 0.03)	10.21 (8.91, 11.71)	9.00 (7.93, 10.18)	9.61 (8.45, 10.83)
Qazvin	7.30 (6.26, 8.40)	5.74 (4.95, 6.59)	6.51 (5.61, 7.43)	0.16 (0.01, 0.73)	0.12 (0.01, 0.56)	0.14 (0.01, 0.65)	0.01 (0.00, 0.03)	0.00 (0.00, 0.02)	0.01 (0.00, 0.02)	7.46 (6.42, 8.58)	5.87 (5.11, 6.71)	6.65 (5.81, 7.58)
Qom	16.08 (13.73, 18.32)	17.54 (15.35, 19.92)	16.75 (14.61, 19.07)	0.32 (0.03, 1.30)	0.36 (0.04, 1.45)	0.34 (0.03, 1.36)	0.01 (0.00, 0.05)	0.01 (0.00, 0.06)	0.01 (0.00, 0.05)	16.41 (14.22, 18.81)	17.91 (15.86, 20.23)	17.10 (14.98, 19.32)
Semnan	10.89 (9.25, 12.46)	10.32 (8.86, 11.88)	10.58 (9.11, 12.14)	0.24 (0.02, 0.97)	0.23 (0.02, 0.93)	0.24 (0.02, 0.94)	0.01 (0.00, 0.04)	0.01 (0.00, 0.03)	0.01 (0.00, 0.04)	11.15 (9.65, 12.64)	10.56 (9.24, 12.07)	10.83 (9.46, 12.37)
Sistan and Baluchistan	32.96 (28.84, 37.38)	30.33 (26.58, 34.32)	31.65 (27.85, 35.35)	0.57 (0.05, 2.51)	0.50 (0.04, 2.14)	0.54 (0.05, 2.33)	0.02 (0.00, 0.10)	0.02 (0.00, 0.08)	0.02 (0.00, 0.09)	33.55 (29.59, 37.86)	30.86 (27.28, 34.88)	32.21 (28.70, 35.98)
South Khorasan	16.20 (14.01, 18.61)	12.88 (11.07, 14.73)	14.51 (12.62, 16.50)	0.31 (0.03, 1.12)	0.24 (0.02, 0.89)	0.28 (0.03, 1.00)	0.01 (0.00, 0.04)	0.01 (0.00, 0.03)	0.01 (0.00, 0.04)	16.53 (14.44, 19.11)	13.13 (11.42, 14.94)	14.80 (12.97, 16.88)
Tehran	16.54 (14.36, 19.37)	21.99 (18.93, 25.37)	19.20 (16.81, 22.07)	0.33 (0.04, 1.17)	0.44 (0.05, 1.63)	0.38 (0.05, 1.39)	0.01 (0.00, 0.04)	0.02 (0.00, 0.06)	0.01 (0.00, 0.05)	16.88 (14.73, 19.66)	22.45 (19.24, 25.69)	19.60 (17.14, 22.36)
West Azarbayejan	9.70 (8.42, 11.11)	7.78 (6.75, 8.91)	8.73 (7.61, 9.93)	0.18 (0.02, 0.70)	0.15 (0.01, 0.55)	0.16 (0.01, 0.63)	0.01 (0.00, 0.03)	0.01 (0.00, 0.02)	0.01 (0.00, 0.02)	9.89 (8.58, 11.27)	7.93 (6.95, 8.99)	8.90 (7.82, 10.01)
Yazd	13.95 (12.04, 16.24)	12.29 (10.57, 14.10)	13.14 (11.38, 14.97)	0.30 (0.03, 1.27)	0.26 (0.02, 1.10)	0.28 (0.03, 1.19)	0.01 (0.00, 0.05)	0.01 (0.00, 0.04)	0.01 (0.00, 0.05)	14.26 (12.35, 16.51)	12.56 (10.98, 14.28)	13.44 (11.77, 15.37)
Zanjan	9.21 (7.88, 10.55)	5.79 (4.93, 6.63)	7.46 (6.46, 8.52)	0.19 (0.02, 0.74)	0.12 (0.01, 0.46)	0.15 (0.01, 0.59)	0.01 (0.00, 0.03)	0.00 (0.00, 0.02)	0.01 (0.00, 0.02)	9.41 (8.19, 10.79)	5.91 (5.12, 6.76)	7.62 (6.67, 8.70)
*Islamic Republic of Iran*	13.64 (12.05, 15.32)	12.82 (11.27, 14.42)	13.24 (11.74, 14.89)	0.26 (0.08, 0.67)	0.25 (0.08, 0.61)	0.26 (0.08, 0.65)	0.01 (0.00, 0.03)	0.01 (0.00, 0.02)	0.01 (0.00, 0.02)	13.92 (12.35, 15.74)	13.08 (11.58, 14.72)	13.51 (11.98, 15.15)

Effective National Tuberculosis Programs (NTPs) that utilize standard four-drug combination therapy and Directly Observed Treatment (DOT) for drug-sensitive tuberculosis achieve cure rates above 95% and significantly reduce the prevalence of tuberculosis strains nationwide. Sustaining these outcomes necessitates ongoing financial and resource investment. Conversely, irregular or underfunded NTPs frequently lead to unsupervised and inadequate treatment, which increases the likelihood of treatment failure and promotes the emergence and transmission of drug resistance. The management of drug-resistant tuberculosis (DR-TB) is substantially more complex than that of drug-sensitive TB and contributes to the accelerated spread of DR-TB [26, 27].

MDR‑TB is defined as resistance to at least isoniazid and Rifampin, the two most potent first‑line anti‑tuberculosis agents. XDR‑TB is characterized by resistance to Rifampin, any fluoroquinolone, and at least one of the newer agents, bedaquiline or linezolid. The management of MDR‑TB and XDR‑TB presents substantial challenges [28]. as treatment regimens often extend for 24 months or longer and depend on second-line drugs, which are typically less effective, more toxic, and associated with a higher risk of adverse effects.

Table 1 presents a detailed analysis of TB cases in Iran in 2021, categorized by sex (male, female, and both combined). The data are further stratified into three clinical classifications: drug‑sensitive TB, MDR‑TB, and XDR‑TB. This table highlights the distribution of treatment sensitivity and resistance across all provinces, offering valuable insights into regional patterns of drug responsiveness and resistance among TB patients. In Iran, the incidence of MDR‑TB is 0.26 per 100,000 population for both sexes [95% confidence interval CI: (0.08–0.65)], while the incidence of XDR‑TB is 0.01 per 100,000 [95% CI: (0.00–0.05)]. These findings indicate that the burden of drug‑resistant TB in Iran remains comparatively low compared to the global average.

**Fig. 2:**
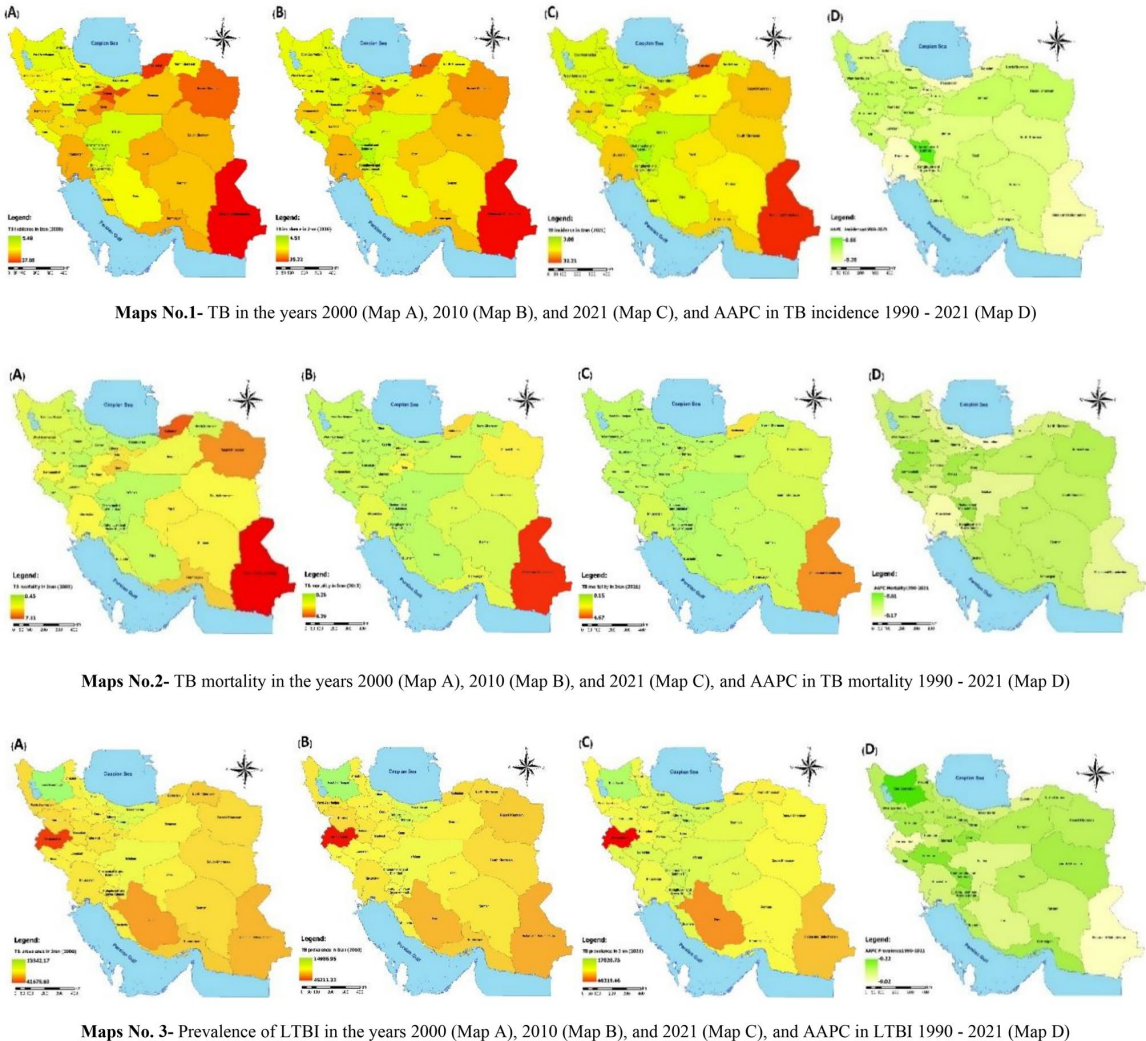
Geographic distribution of incidence, mortality, and prevalence of LTBI and AAPC in Iran, 2000–2021

Figure 2 illustrates the spatial distribution of TB incidence rates in Iran from 2000 to 2021 across both sexes. The maps indicate that TB incidence is disproportionately higher in economically disadvantaged provinces and in provinces bordering Afghanistan and Pakistan, compared with other regions of the country [17]. During the study period, the provinces of Sistan and Baluchistan, Golestan, and Khorasan Razavi consistently reported the highest TB incidence rates. In contrast, the northwestern provinces and central provinces, including Isfahan, Chahar Mahaal and Bakhtiari, and Kohgiluyeh and Boyer-Ahmad, exhibited the lowest incidence rates (Maps 1).

Map 2 illustrates that, despite the continued presence of TB mortality among both sexes in Iran, the national mortality rate was 1.09 per 100,000 population (95% CI: 0.92–1.34) in 2021. Sistan and Baluchistan reported the highest mortality rate at 4.67 per 100,000 (95% CI: 3.35–6.11), whereas Chahar Mahaal and Bakhtiari reported the lowest at 0.15 per 100,000 (95% CI: 0.11–0.26).

Map 3 illustrates the prevalence of LTBI among both sexes. The GBD report indicates that approximately one-quarter of Iran’s population is infected with the TB bacillus. In 2021, the LTBI prevalence rate was estimated at 25,027.92 per 100,000 population (95% CI: 22,356.77–28,068.94) for both sexes. The AAPC for TB incidence, mortality, and LTBI prevalence from 1990 to 2021 was negative, indicating a nationwide decline in these indicators.

**Table 2:** -TB incidence rates and AAPC in the Iranian population 1990–2021

Region	Male				Female			
	2000	2010	2021	AAPC in Male 1990–2021	2000	2010	2021	AAPC in Female 1990–2021
Alborz	13.29 (11.50, 15.23)	12.55 (10.81, 14.31)	12.73 (11.08, 14.62)	-0.74 (-0.96, -0.46)	19.65 (17.15, 22.34)	16.68 (14.58, 18.90)	12.99 (11.34, 14.79)	-1.44 (-1.58, -1.30)
Ardebil	8.17 (7.13, 9.39)	8.86 (7.67, 10.07)	9.31 (8.06, 10.70)	-0.67 (-0.92, -0.42)	10.74 (9.28, 12.30)	9.36 (8.18, 10.70)	8.29 (7.30, 9.38)	-1.67 (-1.81, -1.50)
Bushehr	13.91 (12.16, 15.69)	13.49 (11.81, 15.35)	12.19 (10.56, 13.89)	-0.98 (-1.19, -0.77)	13.64 (11.95, 15.38)	11.85 (10.44, 13.42)	8.71 (7.62, 9.89)	-1.75 (-1.87, -1.61)
Chahar Mahaal and Bakhtiari	5.57 (4.67, 6.47)	4.93 (4.13, 5.77)	3.45 (2.92, 4.05)	-1.89 (-2.02, -1.75)	5.37 (4.65, 6.15)	4.12 (3.52, 4.73)	2.74 (2.34, 3.16)	-2.31 (-2.40, -2.21)
East Azarbayejan	8.07 (5.71, 10.91)	9.25 (7.00, 11.88)	9.44 (7.60, 11.52)	-1.03 (-1.30, -0.73)	10.42 (7.73, 13.44)	9.85 (7.80, 12.49)	8.37 (6.93, 10.05)	-1.68 (-1.84, -1.49)
Fars	10.35 (9.28, 11.56)	9.86 (8.77, 11.06)	10.10 (9.11, 11.26)	-0.89 (-1.15, -0.59)	11.26 (9.96, 12.74)	9.36 (8.27, 10.76)	7.31 (6.46, 8.29)	-1.89 (-2.03, -1.75)
Gilan	13.20 (11.62, 14.98)	12.63 (10.94, 14.41)	11.76 (10.25, 13.51)	-0.85 (-1.03, -0.63)	10.56 (9.24, 12.24)	9.02 (7.90, 10.30)	7.22 (6.30, 8.24)	-1.70 (-1.83, -1.54)
Golestan	30.89 (26.90, 34.83)	29.07 (25.65, 32.96)	28.56 (25.10, 32.46)	-0.72 (-0.94, -0.49)	31.09 (27.42, 35.20)	23.81 (20.76, 26.94)	20.53 (18.06, 23.41)	-1.56 (-1.70, -1.39)
Hamadan	8.06 (7.07, 9.15)	8.47 (7.39, 9.63)	8.16 (7.06, 9.41)	-0.92 (-1.17, -0.64)	9.79 (8.48, 11.06)	8.37 (7.29, 9.54)	6.85 (5.98, 7.82)	-1.76 (-1.89, -1.58)
Hormozgan	20.57 (18.11, 23.43)	20.30 (17.90, 23.02)	19.55 (17.10, 22.26)	-0.81 (-1.05, -0.55)	17.96 (15.96, 20.21)	14.45 (12.85, 16.11)	10.73 (9.42, 12.14)	-1.80 (-1.95, 1.65)
Ilam	9.32 (8.09, 10.73)	8.85 (7.77, 10.20)	8.24 (7.20, 9.49)	-1.13 (-1.34, -0.91)	10.72 (9.32, 12.22)	8.59 (7.49, 9.78)	7.16 (6.26, 8.19)	-1.8 (-1.96, -1.68)
Isfahan	7.01 (6.08, 8.08)	7.14 (6.23, 8.18)	6.88 (5.96, 7.95)	-0.85 (-1.10, -0.60)	9.27 (8.02, 10.77)	8.23 (7.10, 9.43)	6.53 (5.68, 7.44)	-1.67 (-1.81, -1.51)
Kerman	14.98 (13.17, 16.82)	13.31 (11.61, 15.01)	12.71 (11.06, 14.40)	-1.11 (-1.30, -0.88)	17.67 (15.53, 19.86)	14.14 (12.45, 15.87)	11.71 (10.26, 13.29)	-1.61 (-1.75, -1.45)
Kermanshah	15.65 (14.26, 17.15)	15.86 (14.49, 17.27)	16.50 (15.16, 17.93)	-0.95 (-1.22, -0.64)	18.75 (17.16, 20.69)	16.75 (15.06, 18.56)	13.60 (12.37, 15.15)	-1.86 (-1.97, -1.75)
Khorasan-e-Razavi	23.50 (20.58, 26.38)	20.52 (17.95, 23.18)	18.05 (15.91, 20.40)	-1.22 (-1.39, -1.04)	29.28 (25.59, 32.87)	22.46 (19.68, 25.74)	16.59 (14.61, 18.65)	-1.79 (-1.92, 1.64)
Khuzestan	20.00 (17.48, 22.79)	20.25 (17.86, 22.83)	19.71 (17.15, 22.69)	-0.61 (-0.85, -0.34)	17.50 (15.49, 19.83)	16.45 (14.46, 18.71)	13.77 (12.10, 15.67)	-1.21 (-1.37, -1.03)
Kohgiluyeh and Boyer-Ahmad	7.57 (6.57, 8.88)	7.65 (6.66, 8.79)	7.62 (6.60, 8.72)	-0.97 (-1.19, -0.72)	10.32 (8.97, 11.82)	9.43 (8.09, 10.82)	8.22 (7.20, 9.41)	-1.48 (-1.65, -1.32)
Kurdistan	9.30 (8.08, 10.60)	9.24 (8.09, 10.47)	8.46 (7.41, 9.64)	-1.14 (-1.35, -0.90)	14.26 (12.51, 16.18)	11.50 (10.21, 13.02)	8.86 (7.82, 10.02)	-1.86 (-2.00, 1.72)
Lorestan	15.63 (13.59, 17.45)	16.31 (14.17, 18.50)	15.28 (13.34, 17.60)	-0.77 (-1.01, -0.48)	13.16 (11.64, 14.81)	11.47 (10.03, 13.11)	9.75 (8.47, 11.17)	-1.58 (-1.74, -1.42)
Markazi	15.30 (13.44, 17.51)	13.09 (11.52, 15.13)	11.60 (10.10, 13.26)	-1.31 (-1.46, -1.10)	18.25 (16.02, 20.78)	14.32 (12.64, 16.39)	11.17 (9.86, 12.63)	-1.72 (-1.84, -1.56)
Mazandaran	10.24 (8.93, 11.69)	10.77 (9.32, 12.29)	10.18 (8.75, 11.56)	-0.60 (-0.81, -0.35)	8.80 (7.67, 10.18)	8.52 (7.37, 9.92)	7.13 (6.22, 8.22)	-1.39 (-1.55, -1.22)
North Khorasan	10.94 (9.65, 12.38)	10.67 (9.38, 12.08)	10.21 (8.91, 11.71)	-1.04 (-1.23, -0.78)	12.31 (10.90, 14.03)	10.65 (9.37, 12.10)	9.00 (7.93, 10.18)	-1.66 (-1.81, 1.51)
Qazvin	7.91 (6.84, 9.04)	8.10 (7.05, 9.24)	7.46 (6.42, 8.58)	-0.97 (-1.18, -0.71)	9.63 (8.40, 10.94)	7.83 (6.85, 9.02)	5.87 (5.11, 6.71)	-1.93 (-2.05, 1.81)
Qom	19.00 (16.49, 21.55)	18.40 (16.13, 20.74)	16.41 (14.22, 18.81)	-0.97 (-1.20, -0.71)	26.81 (23.62, 29.99)	23.04 (20.36, 25.90)	17.91 (15.86, 20.23)	-1.47 (-1.61, -1.31)
Semnan	15.51 (13.52, 17.57)	13.24 (11.51, 15.17)	11.15 (9.65, 12.64)	-1.31 (-1.45, -1.13)	17.43 (15.28, 19.75)	13.50 (11.90, 15.30)	10.56 (9.24, 12.07)	-1.75 (-1.87, -1.63)
Sistan and Baluchistan	34.53 (31.02, 38.40)	34.83 (30.98, 38.40)	33.55 (29.59, 37.86)	-0.66 (-0.88, -0.44)	39.91 (35.54, 44.94)	35.74 (31.81, 40.31)	30.86 (27.28, 34.88)	-1.31 (-1.48, 1.12)
South Khorasan	18.81 (16.48, 21.32)	18.64 (16.35, 20.87)	16.53 (14.44, 19.11)	-1.02 (-1.24, -0.82)	17.99 (15.89, 20.41)	16.60 (14.57, 18.88)	13.13 (11.42, 14.94)	-1.50 (-1.68, 1.33)
Tehran	22.28 (19.14, 26.00)	19.14 (16.63, 22.02)	16.88 (14.73, 19.66)	-1.24 (-1.43, -1.02)	36.46 (31.56, 42.03)	30.24 (26.57, 34.34)	22.45 (19.24, 25.69)	-1.49 (-1.66, -1.34)
West Azarbayejan	11.58 (10.24, 13.13)	10.28 (9.06, 11.64)	9.89 (8.58, 11.27)	-1.09 (-1.29, -0.87)	13.15 (11.52, 15.04)	10.16 (8.86, 11.70)	7.93 (6.95, 8.99)	-1.84 (-1.97, -1.68)
Yazd	17.75 (15.48, 20.03)	16.49 (14.35, 18.64)	14.26 (12.35, 16.51)	-1.14 (-1.35, -0.94)	19.93 (17.53, 22.69)	16.41 (14.39, 18.53)	12.56 (10.98, 14.28)	-1.64 (-1.77, -1.49)
Zanjan	11.64 (10.16, 13.20)	10.63 (9.33, 12.06)	9.41 (8.19, 10.79)	-1.02 (-1.22, -0.82)	10.08 (8.72, 11.52)	7.86 (6.84, 9.04)	5.91 (5.12, 6.76)	-1.93 (-2.05, 1.78)
Islamic Republic of Iran	15.72 (13.80, 17.70)	14.83 (13.10, 16.58)	13.92 (12.35, 15.74)	-0.97 (-1.11, -0.82)	19.40 (17.30, 21.78)	16.49 (14.72, 18.56)	13.08 (11.58, 14.72)	-1.55 (-1.63, -1.46)

**Fig. 3:**
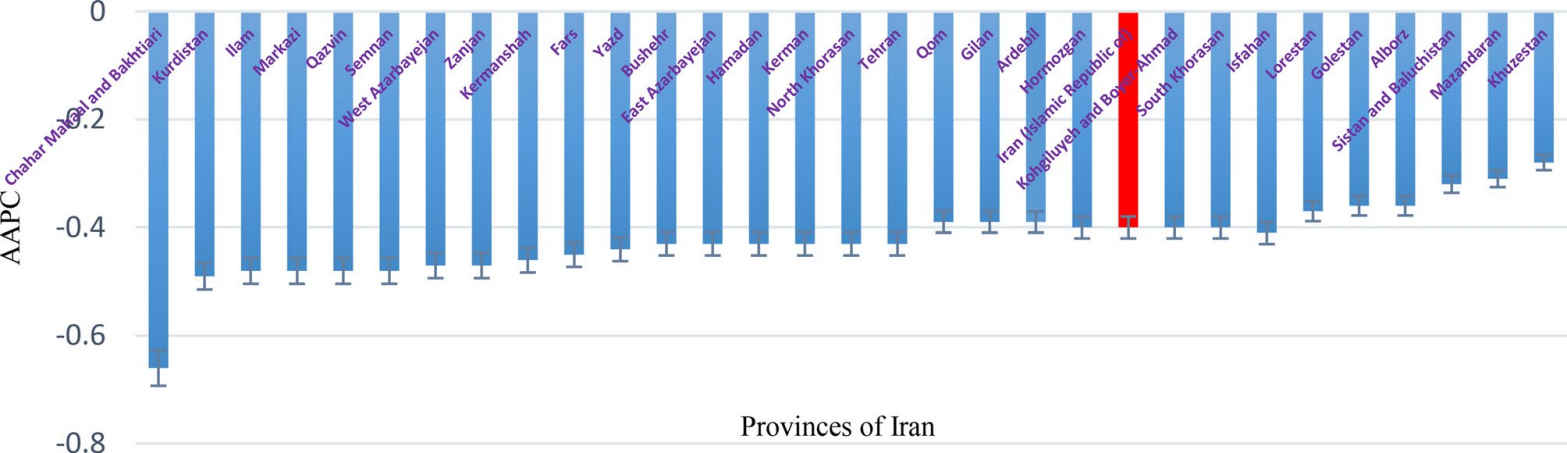
AAPC, TB incidence in both sexes in Iran 1990-2021

Table 2 indicates that the incidence of TB in Iran has consistently declined over recent decades, decreasing from 22.60 per 100,000 population in 1990 to 13.51 per 100,000 in 2021. Figure 3illustrates that the AAPC in TB incidence is negative across all provinces and both sexes. The greatest decline was observed in Chahar Mahaal and Bakhtiari Province, (AAPC–0.66; 95% CI: − 0.69 to − 0.63), whereas the smallest decline occurred in Khuzestan Province, (AAPC: − 0.28; 95% CI: − 0.33 to − 0.22).

**Table 3:** TB mortality rates and the AAPC in the Iranian population, 1990–2021

Region	Male				Female			
	2000	2010	2021	AAPC in Male 1990–2021	2000	2010	2021	AAPC in Female 1990–2021
Alborz	1.68 (1.00, 2.34)	1.16 (0.90, 1.46)	1.05 (0.68, 1.54)	-0.62 (-0.81, 0.25)	1.99 (1.50, 2.48)	1.33 (1.09, 1.58)	1.17 (0.94, 1.48)	-0.64 (-0.74, -0.38)
Ardebil	0.95 (0.62, 1.62)	0.99 (0.83, 1.33)	0.96 (0.69, 1.41)	-0.48 (-0.78, 0.81)	0.97 (0.77, 1.17)	0.59 (0.49, 0.69)	0.55 (0.43, 0.70)	-0.77 (-0.84, -0.61)
Bushehr	2.34 (1.75, 2.89)	1.75 (1.48, 2.05)	1.15 (0.77, 1.74)	-0.68 (-0.84, -0.10)	1.47 (1.22, 1.75)	0.85 (0.72, 0.99)	0.56 (0.44, 0.74)	-0.80 (-0.86, -0.67)
Chahar Mahaal and Bakhtiari	0.55 (0.38, 1.36)	0.36 (0.28, 0.75)	0.23 (0.15, 0.45)	-0.74 (-0.87, -0.25)	0.35 (0.29, 0.44)	0.16 (0.13, 0.19)	0.09 (0.07, 0.13)	-0.88 (-0.91, -0.79)
East Azarbayejan	1.80 (1.32, 2.31)	1.30 (1.09, 1.64)	0.93 (0.67, 1.41)	-0.68 (-0.83, -0.17)	1.63 (1.30, 1.98)	0.83 (0.70, 1.00)	0.62 (0.48, 0.84)	-0.78 (-0.85, -0.63)
Fars	1.42 (1.09, 2.06)	0.94 (0.78, 1.37)	0.70 (0.50, 1.12)	-0.70 (-0.84, -0.21)	0.97 (0.79, 1.15)	0.53 (0.45, 0.63)	0.36 (0.28, 0.51)	-0.78 (-0.85, -0.65)
Gilan	1.85 (1.41, 2.37)	1.40 (1.18, 1.65)	1.07 (0.76, 1.54)	-0.61 (-0.79, -0.01)	0.95 (0.77, 1.12)	0.59 (0.50, 0.68)	0.48 (0.38, 0.59)	-0.70 (-0.79, -0.56)
Golestan	6.48 (4.12, 8.00)	4.38 (2.59, 5.20)	4.15 (2.06, 6.00)	-0.58 (-0.77, 0.10)	4.72 (3.93, 5.55)	2.15 (1.83, 2.51)	2.01 (1.63, 2.48)	-0.76 (-0.83, -0.64)
Hamadan	0.96 (0.68, 1.73)	0.90 (0.74, 1.50)	0.66 (0.48, 1.04)	-0.63 (-0.80, 0.12)	0.82 (0.68, 0.99)	0.53 (0.45, 0.62)	0.42 (0.33, 0.53)	-0.76 (-0.83, -0.63)
Hormozgan	4.30 (3.04, 5.47)	2.86 (2.34, 3.32)	2.12 (1.39, 3.29)	-0.70 (-0.86, -0.12)	2.39 (1.93, 2.78)	1.31 (1.12, 1.51)	0.84 (0.65, 1.12)	-0.82 (-0.87, -0.70)
Ilam	1.37 (1.00, 1.98)	0.93 (0.79, 1.31)	0.83 (0.57, 1.23)	-0.61 (-0.83, 0.20)	1.00 (0.82, 1.18)	0.50 (0.42, 0.58)	0.39 (0.31, 0.52)	-0.80 (-0.88, -0.69)
Isfahan	0.73 (0.54, 1.17)	0.57 (0.47, 0.83)	0.47 (0.33, 0.70)	-0.59 (-0.78, 0.09)	0.70 (0.56, 0.85)	0.43 (0.36, 0.51)	0.39 (0.30, 0.51)	-0.70 (-0.79, -0.53)
Kerman	2.93 (2.28, 3.70)	1.82 (1.54, 2.11)	1.38 (0.99, 2.00)	-0.70 (-0.83, -0.21)	2.28 (1.87, 2.72)	1.17 (0.99, 1.38)	0.91 (0.71, 1.22)	-0.77 (-0.84, -0.63)
Kermanshah	2.90 (2.16, 3.72)	1.81 (1.54, 2.13)	1.23 (0.86, 2.01)	-0.76 (-0.87, -0.32)	2.24 (1.84, 2.65)	1.16 (1.00, 1.33)	0.75 (0.59, 0.98)	-0.82 (-0.87, -0.71)
Khorasan-e-Razavi	4.76 (3.58, 5.91)	3.25 (2.63, 3.78)	2.20 (1.48, 3.37)	-0.72 (-0.85, -0.20)	4.36 (3.61, 5.11)	2.16 (1.84, 2.52)	1.53 (1.18, 2.13)	-0.81 (-0.87, -0.72)
Khuzestan	3.11 (2.20, 3.94)	2.80 (2.13, 3.23)	2.08 (1.20, 3.18)	-0.55 (-0.78, 0.18)	1.96 (1.57, 2.37)	1.23 (1.04, 1.47)	1.05 (0.79, 1.36)	-0.66 (-0.76, -0.45)
Kohgiluyeh and Boyer-Ahmad	0.71 (0.47, 1.35)	0.62 (0.50, 0.85)	0.48 (0.32, 0.79)	-0.62 (-0.83, 0.27)	0.72 (0.55, 0.89)	0.45 (0.37, 0.53)	0.36 (0.28, 0.48)	-0.76 (-0.85, -0.59)
Kurdistan	1.55 (1.12, 2.45)	1.12 (0.91, 1.72)	0.67 (0.46, 1.08)	-0.76 (-0.87, -0.24)	1.66 (1.37, 1.96)	0.93 (0.79, 1.06)	0.56 (0.43, 0.74)	-0.85 (-0.89, -0.75)
Lorestan	2.36 (1.57, 3.21)	2.33 (1.92, 2.74)	1.71 (1.15, 2.53)	-0.56 (-0.81, 0.49)	1.38 (1.09, 1.66)	0.84 (0.70, 0.99)	0.54 (0.40, 0.76)	-0.80 (-0.86, -0.66)
Markazi	2.60 (2.00, 3.21)	1.55 (1.30, 1.83)	1.02 (0.72, 1.56)	-0.76 (-0.87, -0.29)	2.25 (1.84, 2.70)	1.04 (0.86, 1.22)	0.70 (0.53, 0.93)	-0.84 (-0.89, -0.74)
Mazandaran	1.09 (0.75, 1.54)	1.02 (0.84, 1.22)	0.79 (0.56, 1.16)	-0.52 (-0.76, 0.44)	0.58 (0.46, 0.73)	0.44 (0.37, 0.51)	0.39 (0.29, 0.52)	-0.61 (-0.75, -0.36)
North Khorasan	1.68 (1.20, 2.82)	1.37 (1.13, 1.86)	1.07 (0.77, 1.55)	-0.65 (-0.83, 0.10)	1.42 (1.13, 1.77)	0.88 (0.73, 1.05)	0.78 (0.59, 1.02)	-0.75 (-0.83, -0.58)
Qazvin	1.15 (0.78, 1.88)	0.95 (0.79, 1.43)	0.70 (0.50, 1.06)	-0.60 (-0.80, 0.35)	0.89 (0.70, 1.12)	0.50 (0.41, 0.59)	0.34 (0.26, 0.48)	-0.79 (-0.86, -0.66)
Qom	3.25 (2.26, 4.17)	2.48 (1.90, 2.95)	1.53 (0.94, 2.30)	-0.69 (-0.84, -0.02)	3.96 (3.15, 4.70)	2.48 (2.09, 2.94)	1.54 (1.20, 2.05)	-0.76 (-0.83, -0.62)
Semnan	2.65 (2.03, 3.27)	1.70 (1.43, 1.99)	1.18 (0.82, 1.78)	-0.69 (-0.84, -0.10)	2.03 (1.68, 2.42)	1.04 (0.88, 1.22)	0.71 (0.54, 0.98)	-0.80 (-0.86, -0.66)
Sistan and Baluchistan	7.57 (5.46, 9.82)	6.96 (4.69, 8.54)	5.29 (2.70, 7.89)	-0.62 (-0.78, -0.14)	6.65 (5.46, 8.14)	5.60 (4.72, 6.77)	4.03 (3.04, 5.26)	-0.69 (-0.79, -0.52)
South Khorasan	2.89 (2.00, 3.88)	2.63 (1.92, 3.05)	1.81 (1.09, 2.71)	-0.70 (-0.85, -0.01)	2.01 (1.63, 2.54)	1.44 (1.22, 1.70)	1.06 (0.82, 1.43)	-0.79 (-0.85, -0.66)
Tehran	2.48 (1.15, 3.53)	1.63 (0.80, 2.06)	1.07 (0.49, 1.45)	-0.73 (-0.86, -0.17)	3.37 (2.48, 4.20)	1.92 (1.54, 2.35)	1.19 (0.87, 1.61)	-0.80 (-0.87, -0.69)
West Azarbayejan	2.15 (1.60, 2.93)	1.35 (1.15, 1.85)	1.08 (0.75, 1.61)	-0.66 (-0.83, -0.12)	1.63 (1.37, 1.91)	0.71 (0.62, 0.82)	0.61 (0.48, 0.80)	-0.78 (-0.84, -0.66)
Yazd	3.06 (2.22, 3.83)	2.34 (1.91, 2.75)	1.41 (0.89, 2.10)	-0.69 (-0.84, -0.18)	2.35 (1.92, 2.79)	1.26 (1.06, 1.47)	0.87 (0.66, 1.17)	-0.79 (-0.85, -0.64)
Zanjan	1.97 (1.51, 2.44)	1.30 (1.11, 1.58)	0.89 (0.64, 1.35)	-0.66 (-0.82, -0.07)	1.02 (0.84, 1.22)	0.47 (0.38, 0.58)	0.34 (0.25, 0.47)	-0.81 (-0.87, -0.68)
Islamic Republic of Iran	2.46 (1.83, 2.94)	1.78 (1.56, 1.96)	1.28 (1.00, 1.76)	-0.68 (-0.82, -0.19)	2.18 (1.91, 2.40)	1.25 (1.12, 1.38)	0.90 (0.78, 1.07)	-0.77 (-0.81, -0.70)

**Fig. 4:**
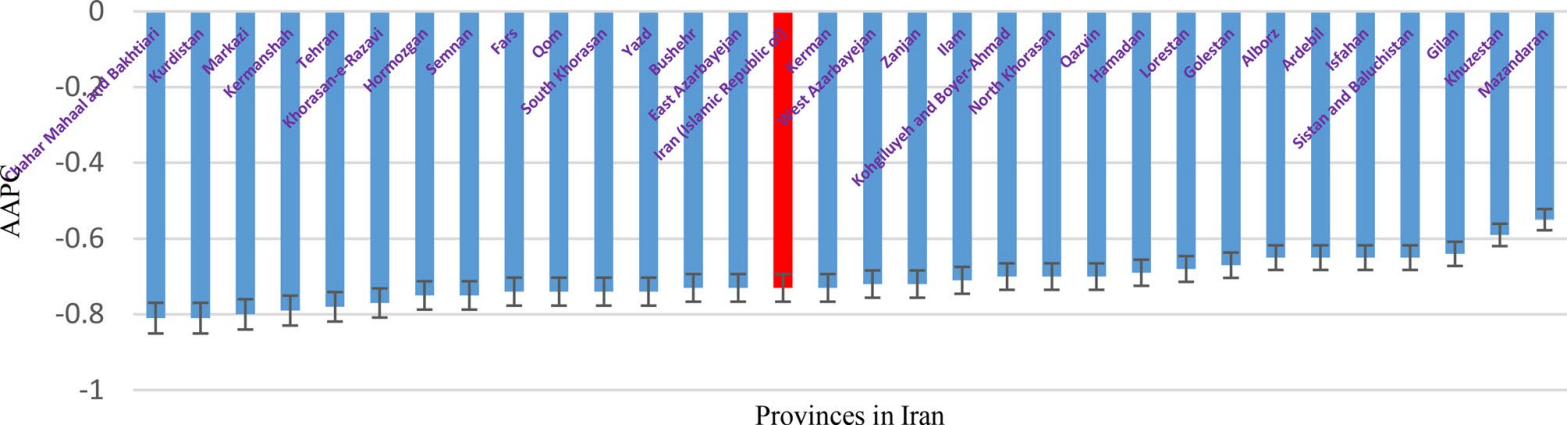
AAPC in TB Mortality in Iran 1990-2021

Table 3 indicates that the TB mortality rate in Iran declined across all provinces between from 1990 to 2021. This reduction occurred in both sexes, with the national mortality rate decreasing from 3.98 per 100,000 population in 1990 to 1.09 per 100,000 in 2021. Figure 4 illustrates that the AAPC in TB mortality also exhibited a negative trend across provinces. The greatest decline was observed in Chahar Mahaal and Bakhtiari [AAPC: − 0.81; 95% CI: (–0.88 to − 0.65)] for both sexes, whereas the smallest decline occurred in Mazandaran Province [AAPC: − 0.55; 95% CI: (–0.72 to − 0.13)].

**Table 4:** Prevalence of LTBI and the AAPC in the Iranian population 1990–2021

		2000			2010			2021			
Region	Male	Female	Both	Male	Female	both	Male	Female	both	(AAPC) Both Sex 1990–2021	
Alborz		24624.37 (21807.06, 27891.66)	24624.37 (21807.06, 27891.66)	25353.40 (22463.42, 28642.45)	23681.96 (20780.93, 26979.56)	23681.96 (20780.93, 26979.56)	24378.70 (21599.04, 27585.51)	21353.11 (18714.41, 24275.82)	21353.11 (18714.41, 24275.82)	22319.77 (19604.55, 25322.43)	-0.37 (-0.49, -0.23)
Ardebil		26659.92 (23567.17, 29895.36)	26659.92 (23567.17, 29895.36)	27397.50 (24568.39, 30717.48)	26096.22 (23011.31, 29496.20)	26096.22 (23011.31, 29496.20)	26866.78 (23701.38, 30219.15)	23843.45 (20996.61, 27223.25)	23843.45 (20996.61, 27223.25)	24933.47 (22154.20, 28148.29)	-0.42 (-0.59, -0.25)
Bushehr		26324.09 (23377.92, 29594.33)	26324.09 (23377.92, 29594.33)	27071.33 (24349.95, 30275.10)	26711.03 (23738.69, 30356.09)	26711.03 (23738.69, 30356.09)	27423.09 (24460.52, 31142.62)	22966.74 (19985.29, 26145.57)	22966.74 (19985.29, 26145.57)	24049.40 (21152.92, 27149.99)	-0.40 (-0.56, -0.23)
Chahar Mahaal and Bakhtiari		25308.64 (22481.00, 28677.97)	25308.64 (22481.00, 28677.97)	26073.40 (23217.89, 29342.39)	24494.57 (21620.83, 28030.48)	24494.57 (21620.83, 28030.48)	25253.86 (22384.71, 28570.77)	20930.86 (18373.32, 23920.77)	20930.86 (18373.32, 23920.77)	21964.91 (19320.87, 24927.20)	-0.53 (-0.67, -0.37)
East Azarbayejan		12396.31 (8668.50, 18034.66)	12396.31 (8668.50, 18034.66)	13342.17 (9574.19, 18776.05)	14259.30 (10566.71, 19086.78)	14259.30 (10566.71, 19086.78)	14986.95 (11267.48, 19757.22)	16084.80 (13068.97, 19865.33)	16084.80 (13068.97, 19865.33)	17008.43 (13999.06, 20730.53)	-0.70 (-0.88, -0.51)
Fars		35028.40 (32061.45, 37963.78)	35028.40 (32061.45, 37963.78)	34329.34 (31408.75, 37347.69)	33905.29 (30856.05, 37048.06)	33905.29 (30856.05, 37048.06)	32497.54 (29549.23, 35658.99)	36824.92 (33832.54, 40104.08)	36824.92 (33832.54, 40104.08)	35531.21 (32545.43, 38862.23)	-0.18 (-0.37, -0.01)
Gilan		24768.85 (21861.53, 27995.51)	24768.85 (21861.53, 27995.51)	25523.84 (22637.19, 28760.19)	24471.76 (21678.53, 27945.42)	24471.76 (21678.53, 27945.42)	25231.64 (22572.07, 28533.08)	21961.94 (19271.88, 24885.84)	21961.94 (19271.88, 24885.84)	23043.20 (20265.48, 26005.09)	-0.36 (-0.50, -0.20)
Golestan		28649.97 (25715.49, 32081.65)	28649.97 (25715.49, 32081.65)	29402.11 (26617.89, 32541.94)	28603.47 (25626.20, 32066.44)	28603.47 (25626.20, 32066.44)	29370.51 (26343.03, 32999.95)	26358.42 (23460.70, 29702.94)	26358.42 (23460.70, 29702.94)	27454.73 (24493.74, 30849.89)	-0.23 (-0.41, -0.06)
Hamadan		27447.15 (24529.11, 30888.43)	27447.15 (24529.11, 30888.43)	28224.18 (25261.71, 31635.86)	27668.00 (24457.10, 31084.06)	27668.00 (24457.10, 31084.06)	28402.05 (25266.08, 31871.44)	24924.48 (21832.97, 28404.46)	24924.48 (21832.97, 28404.46)	25989.76 (23057.08, 29410.20)	-0.30 (-0.45, -0.14)
Hormozgan		29802.21 (26918.79, 33432.76)	29802.21 (26918.79, 33432.76)	30523.63 (27619.10, 33944.24)	29140.92 (25936.88, 32673.37)	29140.92 (25936.88, 32673.37)	29845.75 (26950.98, 33362.36)	25198.37 (22305.41, 28528.80)	25198.37 (22305.41, 28528.80)	26239.62 (23395.42, 29637.36)	-0.45 (-0.62, -0.26)
Ilam		26408.07 (23425.54, 29731.59)	26408.07 (23425.54, 29731.59)	27145.95 (24213.81, 30545.90)	25852.38 (22949.50, 29370.79)	25852.38 (22949.50, 29370.79)	26609.13 (23612.71, 29866.16)	23252.13 (20333.41, 26634.91)	23252.13 (20333.41, 26634.91)	24306.93 (21364.39, 27408.38)	-0.38 (-0.54, -0.21)
Isfahan		24414.62 (21455.58, 27848.17)	24414.62 (21455.58, 27848.17)	25199.27 (22345.98, 28505.34)	24781.89 (21766.98, 28298.08)	24781.89 (21766.98, 28298.08)	25484.06 (22644.79, 28699.97)	22260.59 (19598.66, 25486.45)	22260.59 (19598.66, 25486.45)	23327.75 (20621.24, 26486.74)	-0.26 (-0.40, -0.10)
Kerman		28236.77 (25205.18, 31578.10)	28236.77 (25205.18, 31578.10)	29015.00 (26072.42, 32252.81)	27715.95 (24634.18, 31255.14)	27715.95 (24634.18, 31255.14)	28455.25 (25391.63, 31943.99)	25147.43 (22216.46, 28373.96)	25147.43 (22216.46, 28373.96)	26201.40 (23202.65, 29399.78)	-0.32 (-0.47, -0.16)
Kermanshah		41508.68 (39079.00, 44275.46)	41508.68 (39079.00, 44275.46)	41678.83 (39284.56, 44236.08)	45583.37 (42438.13, 48379.16)	45583.37 (42438.13, 48379.16)	45211.33 (41977.38, 47922.34)	46569.50 (43753.57, 49327.36)	46569.50 (43753.57, 49327.36)	46296.20 (43450.78, 48888.54)	-0.10 (-0.21, -0.01)
Khorasan-e-Razavi		29054.72 (26145.18, 32413.87)	29054.72 (26145.18, 32413.87)	29777.43 (26958.61, 33165.80)	29157.14 (25774.24, 32605.75)	29157.14 (25774.24, 32605.75)	29916.13 (26513.66, 33300.15)	25300.91 (22455.06, 28683.37)	25300.91 (22455.06, 28683.37)	26392.13 (23601.15, 29748.58)	-0.39 (-0.56, -0.20)
Khuzestan		27503.23 (24477.13, 30988.12)	27503.23 (24477.13, 30988.12)	28287.15 (25320.91, 31714.36)	27495.79 (24240.36, 30934.22)	27495.79 (24240.36, 30934.22)	28218.13 (25077.98, 31567.92)	24212.17 (21364.27, 27429.50)	24212.17 (21364.27, 27429.50)	25319.91 (22575.61, 28585.02)	-0.28 (-0.43, -0.12)
Kohgiluyeh and Boyer-Ahmad		26217.29 (23192.85, 29746.97)	26217.29 (23192.85, 29746.97)	26940.39 (24057.67, 30220.54)	25194.88 (22227.36, 28689.75)	25194.88 (22227.36, 28689.75)	25916.50 (23050.71, 29258.29)	22182.33 (19442.23, 25285.24)	22182.33 (19442.23, 25285.24)	23230.31 (20365.68, 26311.21)	-0.48 (-0.62, -0.32)
Kurdistan		28314.44 (25179.40, 31646.73)	28314.44 (25179.40, 31646.73)	29066.47 (26151.81, 32263.59)	28132.24 (25056.94, 31782.32)	28132.24 (25056.94, 31782.32)	28837.64 (25888.33, 32432.44)	24098.64 (21234.30, 27375.07)	24098.64 (21234.30, 27375.07)	25190.28 (22374.53, 28573.62)	-0.46 (-0.62, -0.30)
Lorestan		26980.00 (23956.83, 30183.87)	26980.00 (23956.83, 30183.87)	27742.52 (24870.94, 30955.19)	25896.04 (22885.75, 29555.26)	25896.04 (22885.75, 29555.26)	26650.68 (23593.62, 30236.62)	22410.92 (19747.13, 25620.94)	22410.92 (19747.13, 25620.94)	23516.10 (20825.77, 26796.57)	-0.55 (-0.72, -0.38)
Markazi		27017.17 (24010.73, 30567.46)	27017.17 (24010.73, 30567.46)	27825.97 (24916.37, 31222.63)	26736.04 (23610.11, 30439.51)	26736.04 (23610.11, 30439.51)	27513.21 (24541.08, 30972.44)	23501.08 (20420.78, 26854.17)	23501.08 (20420.78, 26854.17)	24593.70 (21651.68, 27781.79)	-0.41 (-0.56, -0.25)
Mazandaran		23739.44 (20901.51, 26974.86)	23739.44 (20901.51, 26974.86)	24502.68 (21753.57, 27701.76)	23698.52 (20858.08, 26887.52)	23698.52 (20858.08, 26887.52)	24430.23 (21652.43, 27465.61)	21398.03 (18585.25, 24393.32)	21398.03 (18585.25, 24393.32)	22452.63 (19665.13, 25499.15)	-0.28 (-0.43, -0.13)
North Khorasan		29553.72 (26665.28, 32819.26)	29553.72 (26665.28, 32819.26)	30235.55 (27345.81, 33347.04)	28797.49 (25510.47, 32573.38)	28797.49 (25510.47, 32573.38)	29571.07 (26576.54, 33044.32)	25982.76 (22992.57, 29478.19)	25982.76 (22992.57, 29478.19)	27079.60 (23986.25, 30624.52)	-0.40 (-0.59, -0.20)
Qazvin		26733.79 (23690.45, 30212.68)	26733.79 (23690.45, 30212.68)	27507.53 (24612.64, 30773.24)	26620.97 (23684.25, 30081.34)	26620.97 (23684.25, 30081.34)	27373.05 (24429.24, 30832.02)	23422.99 (20534.36, 26690.09)	23422.99 (20534.36, 26690.09)	24483.16 (21583.35, 27727.39)	-0.42 (-0.57, -0.26)
Qom		27386.48 (24353.83, 30936.84)	27386.48 (24353.83, 30936.84)	28159.32 (25197.89, 31561.73)	26499.24 (23694.34, 30111.05)	26499.24 (23694.34, 30111.05)	27238.26 (24452.71, 30684.78)	22618.57 (19908.69, 25789.87)	22618.57 (19908.69, 25789.87)	23640.54 (20927.17, 26865.66)	-0.52 (-0.65, -0.35)
Semnan		26452.41 (23385.49, 29938.76)	26452.41 (23385.49, 29938.76)	27212.02 (24303.07, 30539.64)	26179.62 (23148.88, 29850.19)	26179.62 (23148.88, 29850.19)	26958.50 (24034.04, 30475.20)	23143.80 (20268.49, 26453.98)	23143.80 (20268.49, 26453.98)	24221.82 (21405.99, 27447.38)	-0.40 (-0.54, -0.24)
Sistan and Baluchistan		31716.03 (28803.52, 34905.94)	31716.03 (28803.52, 34905.94)	32356.82 (29456.87, 35164.39)	33069.93 (29976.29, 36413.38)	33069.93 (29976.29, 36413.38)	33699.94 (30692.38, 36774.43)	30294.87 (27059.78, 33785.13)	30294.87 (27059.78, 33785.13)	31242.37 (28292.48, 34568.01)	-0.06 (-0.21, 0.10)
South Khorasan		28350.37 (25579.28, 31550.32)	28350.37 (25579.28, 31550.32)	29129.54 (26212.84, 32338.90)	28127.35 (25062.48, 31578.03)	28127.35 (25062.48, 31578.03)	28903.59 (25769.05, 32407.19)	24649.49 (21841.41, 28062.60)	24649.49 (21841.41, 28062.60)	25715.42 (22857.35, 28867.01)	-0.46 (-0.64, -0.30)
Tehran		22668.08 (19853.44, 25869.77)	22668.08 (19853.44, 25869.77)	23457.81 (20602.73, 26584.28)	22358.93 (19738.26, 25517.49)	22358.93 (19738.26, 25517.49)	23081.15 (20392.35, 26198.65)	19985.83 (17437.86, 23150.64)	19985.83 (17437.86, 23150.64)	20977.29 (18327.57, 24084.20)	-0.38 (-0.52, -0.23)
West Azarbayejan		28784.01 (25708.63, 32346.05)	28784.01 (25708.63, 32346.05)	29498.01 (26624.84, 32915.09)	27946.34 (24875.84, 31270.51)	27946.34 (24875.84, 31270.51)	28732.65 (25551.76, 32099.64)	24738.78 (21804.46, 27949.80)	24738.78 (21804.46, 27949.80)	25805.17 (22973.61, 28913.72)	-0.38 (-0.55, -0.21)
Yazd		26044.22 (23058.40, 29438.37)	26044.22 (23058.40, 29438.37)	26840.98 (23976.66, 30114.46)	26291.23 (23294.65, 29473.35)	26291.23 (23294.65, 29473.35)	27009.10 (24068.21, 30152.16)	22768.94 (19992.60, 26210.97)	22768.94 (19992.60, 26210.97)	23823.72 (20966.83, 27058.28)	-0.40 (-0.57, -0.25)
Zanjan		26939.79 (23893.18, 30616.53)	26939.79 (23893.18, 30616.53)	27730.82 (24805.75, 31183.30)	26458.24 (23433.59, 30003.31)	26458.24 (23433.59, 30003.31)	27214.40 (24277.41, 30583.55)	23161.55 (20214.08, 26335.06)	23161.55 (20214.08, 26335.06)	24273.60 (21385.69, 27442.55)	-0.38 (-0.52, -0.19)
Islamic Republic of Iran		26394.33 (23772.70, 29402.06)	26394.33 (23772.70, 29402.06)	27067.24 (24431.21, 30117.92)	26411.26 (23710.59, 29468.76)	26411.26 (23710.59, 29468.76)	27000.95 (24240.57, 30108.33)	24158.29 (21602.05, 27084.92)	24158.29 (21602.05, 27084.92)	25027.92 (22356.77, 28068.94)	-0.35 (-0.44, -0.26)

Table 4 shows that the prevalence of LTBI in Iran gradually declined from 1990 to 2021. The estimated number of cases remained relatively stable between 2000 and 2010, followed by a marked reduction by 2021. This downward trend is further supported by an AAPC of − 0.35%, indicating a consistent, albeit modest, decrease in LTBI prevalence across the population. This decline likely reflects improvements in public health interventions, TB control programs, and early detection strategies implemented nationwide.

**Fig. 5:**
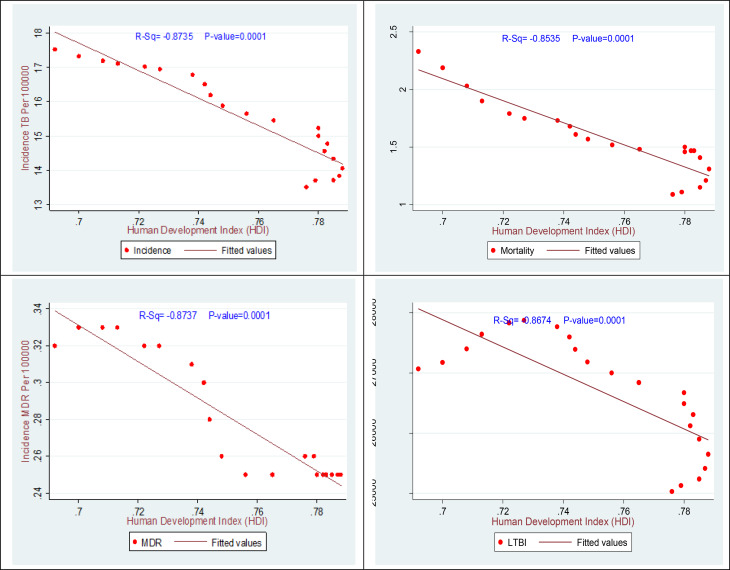
AAPC in the Prevalence of (LTBI) in Iran 1990-2021

The HDI is a composite measure that ranks countries on human development, across three primary dimensions: health, education, and living standards. This index incorporates indicators such as life expectancy, educational attainment, and per capita income, providing a comprehensive assessment of development that extends beyond economic growth. The HDI scale ranges from 0 to 1. In 2021, Iran’s HDI was 0.780 (Global Data Lab, https://globaldatalab.org/shdi/download/shdi/IRN/) [18] Notable provincial disparities, exist with Tehran and Alborz recording the highest HDI (0.817), whereas Sistan and Baluchestan reported the lowest (0.671) (Fig 5).

**Fig. 6:**
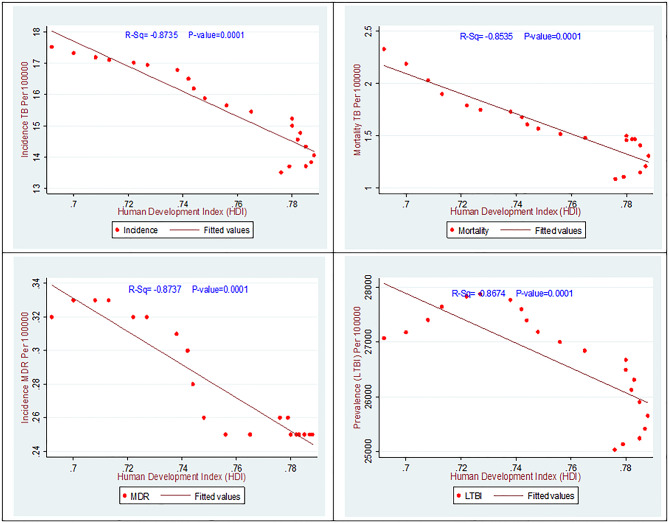
The association between the HDI and TB incidence, mortality, MDR, and LTBI in Iran 2000–2021

Previous studies have demonstrated associations between disease incidence, mortality, prevalence, MDR-TB, and the HDI, indicating that these relationships may be direct, indirect, or statistically significant. In the present study (Fig. 6), a strong, statistically significant inverse association was observed among TB incidence, TB-related mortality, and HDI. Moreover, a very strong inverse association between latent tuberculosis infection LTBI, MDR-TB, and HDI was also statistically significant (*P* < 0.05). Thus, higher HDI values are associated with significantly lower incidence and prevalence of TB, TB-related mortality, MDR-TB, and LTBI (*P* < 0.05).

## Discussion

According to GBD data, age-standardized incidence rate (ASIR) of TB in Iran declined from 22.60 cases per 100,000 population (95% CI: 20.09–25.41) in 1990 to 13.51 cases (95% CI: 11.98–15.15) in 2021. In 2021, the TB mortality rate was 1.09 cases per 100,000 population (95% CI: 0.92–1.34). This reduction is attributted to the implementation of TB control measures, including the Directly Observed Treatment, Short-course (DOTS) strategy, expanded Bacillus Calmette–Guérin (BCG) vaccine coverage, improved diagnostic and treatment infrastructure, and increased public awareness. These findings align with Khazaei et al. (2016), who reported reductions in TB incidence, prevalence, and mortality in Iran from 1990 to 2014 [29]. Globally, analyses of TB incidence trends from 2000 to 2021 demonstrate an overall decline. However, Iran’s incidence rate of 13.51 cases per 100,000 population remains substantially lower than the average reported for middle-income countries (115 cases per 100,000) [27]. The GBD 2021 report indicates that reductions in certain regions, including the Middle East, have been less pronounced than the global average. Notably, Iran outperformed several neighboring countries in TB control [29].

Significant geographical variation in TB patterns is evident across provinces. Border regions, notably Sistan and Baluchestan (32.21 cases per 100,000 population) and Golestan (24.46 cases per 100,000), report the highest incidence and prevalence rates. These elevated burdens are likely attributable to contextual factors, including proximity to high-risk countries, cross-border migration, and limited access to healthcare services [17].

Provinces such as Chahar Mahaal and Bakhtiari (3.08 cases per 100,000 population) and Isfahan (6.71 cases per 100,000) reported the lowest TB rates. These disparities are plausibly associated with contextual factors, including proximity to high-incidence countries such as Afghanistan and Pakistan, with TB incidence rates of 189 and 263 cases per 100,000 population, respectively Additional contributing factors include migration, poverty, restricted healthcare access, and broader socioeconomic conditions. For instance, Sistan and Baluchestan, which is characterized by a low (HDI: 0.671) and high poverty levels, exhibited the highest TB mortality rate (4.67 cases per 100,000 population) In contrast, more developed provinces such as Tehran (HDI: 0.817) demonstrated lower rates. These findings are consistent with those of Kiani et al. (2021), who identified high-risk TB clusters in southeastern and northeastern Iran [30]. Additional domestic studies, including the analysis of the spatial distribution of smear-positive pulmonary TB incidence rates in Iran from 2018 to 2022, corroborate the presence of high-risk clusters along the eastern borders and further document an overall decline, albeit with persistent regional disparities [28].

Epidemiological analyses of TB in Middle Eastern countries indicate that Iran has lower incidence rates compared to neighboring countries, including Afghanistan. Border clusters in Iran exhibit patterns similar to those found in areas affected by African migration [31]. A report from southern Iranian provinces demonstrates a 34% reduction in TB incidence, consistent with the national trend, but also highlights ongoing provincial disparities [32]. There is a statistically significant inverse correlation (*P* < 0.05) between TB indicators and the HDI. Provinces with higher HDI, such as Tehran and Alborz, characterized by greater life expectancy, higher educational attainment, and increased per capita income, have experienced more rapid declines in TB incidence and mortality.

According to United Nations Development Programme (UNDP) data, Iran’s HDI increased from 0.57 in 1990 to 0.78 in 2021, which may have contributed to the observed reduction in TB burden [17, 33]. This improvement is plausibly attributable to enhanced healthcare access, improved nutrition, higher literacy rates, and increased public awareness, all of which serve as protective factors against TB [17, 34]. In contrast, provinces with lower HDI values, such as Sistan and Baluchestan and Golestan, continue to face persistent challenges in TB control This likely reflects, the impact of socioeconomic disparities on public health outcomes. These findings are consistent with those of Fallahzadeh et al. (2023), who analyzed TB incidence and mortality distributions in Iran using GBD data 2019 data and reported a strong inverse correlation with HDI [17, 35, 36].

Additional domestic studies, including analyses of TB incidence and mortality distributions in relation to the HDI in Iran from 2010 to 2019, confirm a significant negative correlation (*r* = − 0.36) and further document reductions in high-HDI provinces [17, 30]. At the global level, research examining the impact of the HDI on TB incidence in Asia has demonstrated a significant negative correlation (*R* = − 0.609) in Southeast Asian countries, which aligns with the present findings [37, 38]. Conversely, global studies analyzing TB incidence disparities by HDI indicate that TB incidence in low-HDI countries is approximately six times higher, reflecting patterns similar to the provincial disparities observed in Iran but on a broader scale [27].

This study, examined the effects of age and birth cohort on TB incidence and mortality. The results demonstrated a pronounced age effect, as both incidence and mortality rates increased with advancing age. This trend is likely due to age-related declines in immune function., the increased prevalence of comorbidities, and reduced access to healthcare services among older populations. Furthermore, cohort analysis indicated that more recent birth cohorts exhibit lower rates of TB incidence and mortality than earlier cohorts. This reduction likely results from improvements in socioeconomic conditions, increased public health awareness, and the effectiveness of national TB control programs over the past few decades [39].

The average annual percent change (AAPC) for tuberculosis (TB) incidence (–0.40) and mortality (–0.73) indicates a sustained downward trend. The cohort effect reveals generational disparities: individuals born before 1960 experience higher TB rates, likely due to increased childhood exposure and limited access to contemporary treatments. In contrast, those born after 1990 benefit from vaccination programs and socioeconomic improvements. Further domestic studies, such as analyses of TB incidence trends in Khuzestan Province, Iran (2010–2021), reinforce the importance of age and period effects and predict a continued decline in TB incidence through 2050 [17, 28, 40–42].

Despite significant advancements, multidrug-resistant tuberculosis (MDR-TB) and extensively drug-resistant tuberculosis (XDR-TB) remain significant challenges. In Iran, the incidence of MDR-TB is 0.26 cases per 100,000 population, and XDR-TB is 0.01 cases per 100,000, both of which are below the global average. However, the prevalence of MDR-TB among newly diagnosed cases, at approximately 1.7%, continues to pose a serious public health concern [17, 43].

Incomplete treatment courses, diagnostic limitations, and cross-border transmission have been identified as contributing factors [17]. The high prevalence of latent tuberculosis infection (LTBI), estimated at 25,027 cases per 100,000 population and particularly elevated in provinces such as Kermanshah (46,296 cases), represents a significant reservoir for future active tuberculosis cases. This highlights the necessity of targeted screening and preventive treatment for LTBI among high-risk populations, especially in border regions. These results align with those of Ayoubi et al. (2024), who reported a multidrug-resistant tuberculosis (MDR-TB) prevalence of 0.6% in Iran [44].

Global analyses of MDR-TB prevalence report a rate of 11.6%, which exceeds the rate observed in Iran and highlights a comparable upward trend in developing countries [40]. Investigations into the prevalence of LTBI among Iranian healthcare workers, which range from 2% to 49%, also indicate higher rates in high-risk provinces, thereby supporting the provincial patterns identified in the present study [40].

### Limitations

Several important limitations are present in this study. The ecological design limits causal inference, and reliance on Global Burden of Disease (GBD) data may reduce precision in regions with limited surveillance. The complexity of age–period–cohort (APC) modeling also complicates the separation of overlapping temporal effects. However, the combined application of APC and Human Development Index (HDI) analyses offers a comprehensive perspective for health policy, and domestic evaluations have generally affirmed the reliability of GBD estimates [40].

Other limitations include the lack of individual-level and contextual data, such as household size, occupational status, comorbidities, and urban–rural differences, as well as behavioral and environmental exposures, including nutrition, smoking, and HIV status. Health system factors, such as access to diagnostics, treatment adherence, and variability in surveillance infrastructure, were also not captured. Additionally, reporting biases may introduce uncertainty. Although these omissions limit the granularity of the analysis, they do not compromise the validity of GBD data as the most comprehensive source for global comparative assessments.

## Conclusion

The findings indicate that Iran has achieved significant reductions in the incidence, mortality, and prevalence of tuberculosis (TB) from 1990 to 2021. National TB rates remain below the global average, with a consistent downward trend observed across all provinces. However, regional disparities persist in border provinces such as Sistan and Baluchistan and Golestan, and ongoing challenges related to multidrug-resistant TB (MDR-TB) and latent TB infection (LTBI) continue. The inverse correlation between the Human Development Index (HDI) and TB indicators highlights the importance of socioeconomic development in disease control. To accelerate progress toward the global End TB Strategy targets by 2030, targeted interventions are required, including migrant screening, enhanced diagnosis and treatment of MDR-TB, and expanded preventive therapy for LTBI in high-risk regions.

## Data Availability

The datasets analyzed in this study can be found from the Global Health DataExchange (GHDx) website https://vizhub.healthdata.org/gbd-results/.

## References

[1] Zhang S-X, et al. Epidemiological features and temporal trends of the co-infection between HIV and tuberculosis, 1990–2021: findings from the Global Burden of Disease Study 2021. Infect Dis poverty. 2024;13(04):79–94. 39152514 10.1186/s40249-024-01230-3 PMC11328430

[2] Yang H, et al. Global, regional, and national burden of tuberculosis and attributable risk factors for 204 countries and territories, 1990–2021: A systematic analysis for the Global Burden of Diseases 2021 study. BMC Public Health. 2024;24(1):3111. 39529028 10.1186/s12889-024-20664-w PMC11552311

[3] Al Maani A, Petersen E, Memish ZA. The critical role of new tuberculosis vaccines in achieving the WHO 2035 End TB target. IJID Reg. 2025;14:100595. 40201559 10.1016/j.ijregi.2025.100595 PMC11973680

[4] Chunrong L, et al. 2023 WHO Tuberculosis Report: key data analysis for China and the global world. Electron J Emerg Infect Dis. 2023;8(6):73.

[5] Zhang S-X, et al. Global, regional, and National burden of HIV-negative tuberculosis, 1990–2021: findings from the global burden of disease study 2021. Infect Dis Poverty. 2024;13(1):60. 39155365 10.1186/s40249-024-01227-y PMC11331668

[6] Godoy P. Guidelines on controlling latent tuberculosis infection to support tuberculosis elimination. Revista Española de Sanidad Penitenciaria. 2021;23(1):28. 33847703 10.18176/resp.00028 PMC8278168

[7] Liebenberg D, Gordhan BG, Kana BD. Drug resistant tuberculosis: Implications for transmission, diagnosis, and disease management. Front Cell Infect Microbiol. 2022;12:943545. 36211964 10.3389/fcimb.2022.943545 PMC9538507

[8] Naidoo K, et al. The epidemiology, transmission, diagnosis, and management of drug-resistant tuberculosis—lessons from the South African experience. Lancet Infect Dis. 2024;24(9):e559–75. 38527475 10.1016/S1473-3099(24)00144-0

[9] Gilmour B, Alene KA. Ending tuberculosis: Challenges and opportunities. Front Tuberculosis. 2024;2:1487518. 10.3389/ftubr.2024.1487518 PMC13495007 42638666

[10] Költringer FA, et al. The social determinants of national tuberculosis incidence rates in 116 countries: a longitudinal ecological study between 2005–2015. BMC Public Health. 2023;23(1):337. 36793018 10.1186/s12889-023-15213-w PMC9930041

[11] Okram M, Singh OM. Tuberculosis: A narrative review on epidemiology, risks, implications, preventions and treatments. Int J Res Med Sci. 2024;12(6):2172.

[12] Matteelli A, et al. Update on multidrug-resistant tuberculosis preventive therapy toward the global tuberculosis elimination. Int J Infect Dis. 2025;155. 10.1016/j.ijid.2025.107849 39993523

[13] Kudryavtsev I, et al. The role of the immune response in developing tuberculosis infection: from latent infection to active tuberculosis. Front Tuberculosis. 2024;2:1438406. 10.3389/ftubr.2024.1438406 PMC13494988 42638763

[14] Martinez L, et al. Effectiveness of preventive treatment among different age groups and Mycobacterium tuberculosis infection status: a systematic review and individual-participant data meta-analysis of contact tracing studies. Lancet Respiratory Med. 2024;12(8):633–41. 10.1016/S2213-2600(24)00083-3 PMC12061052 38734022

[15] Clark RA. Mathematical modelling of the impact of adolescent/adult tuberculosis vaccines to inform global, national, and subnational policy and delivery. London School of Hygiene & Tropical Medicine; 2023.

[16] Collins JM, et al. Prevalence of latent tuberculosis infection among non-US-born persons by country of birth—United States, 2012–2017. Clin Infect Dis. 2021;73(9):e3468–75. 33137172 10.1093/cid/ciaa1662 PMC8563169

[17] Fallahzadeh H, et al. Distribution incidence, mortality of tuberculosis and human development index in Iran: estimates from the global burden of disease study 2019. BMC Public Health. 2023;23(1):2404. 38049770 10.1186/s12889-023-17114-4 PMC10694928

[18] Negintaji Z, Esmailinia S. Calculation of the Economic Globalization Index in Iran and its Impact on Human Development Index (HDI). Macroeconomics Res Letter. 2024;18(40):153–77.

[19] Mariani F, Ciommi M. Aggregating composite indicators through the geometric mean: A penalization approach. Computation. 2022;10(4):64.

[20] Mehmetoglu M, Jakobsen TG. Applied statistics using Stata: a guide for the social sciences. Sage; 2022.

[21] Khazaei Z, et al. Global incidence and mortality of esophageal cancer and its relationship with the Human Development Index (HDI); An Ecology Study. Asian Pac J Cancer Nurs, 2019: pp. 20191111–20191111.

[22] Tan E-L, et al. Global burden of MDR-TB and XDR-TB: trends, inequities, and future implications for public health planning. BMC Infect Dis. 2025;25(1):1225. 41039228 10.1186/s12879-025-11566-2 PMC12490063

[23] Song H-W, et al. Tracking multidrug resistant tuberculosis: a 30-year analysis of global, regional, and national trends. Front Public Health. 2024;12:1408316. 39319291 10.3389/fpubh.2024.1408316 PMC11421170

[24] Alinaghian SA, et al. Burden of type 2 diabetes and its relationship with human development index in Asian countries: Global Burden of Disease Study in 2019. BMC Public Health. 2025;25(1):402. 39891143 10.1186/s12889-025-21608-8 PMC11786590

[25] Khazaei Z et al. Disability-adjusted life years of Hepatitis B in Iran during 2009–2019: an analysis based on the global burden of Disease Study 2019. Open Public Health J. 2024;17(1).

[26] Elbehiry A, et al. Advancing the fight against tuberculosis: integrating innovation and public health in diagnosis, treatment, vaccine development, and implementation science. Front Med. 2025;12:1596579. 10.3389/fmed.2025.1596579 PMC12415020 40927186

[27] Bai W, Ameyaw EK. Global, regional and national trends in tuberculosis incidence and main risk factors: a study using data from 2000 to 2021. BMC Public Health. 2024;24(1):12. 38166735 10.1186/s12889-023-17495-6 PMC10759569

[28] Rastegar M, et al. Spatial Distribution of Smear-Positive Pulmonary Tuberculosis Incidence Rates in Iran: A Registry-Based Study (2018–2022). Int J Prev Med. 2024;15:35. 39239301 10.4103/ijpvm.ijpvm_346_23 PMC11376526

[29] Khazaei S, Mansournia AE, Rafiemanesh MA. H., Trend of some Tuberculosis Indices in Iran during 25 year Period (1990–2014). J Res Health Sci. 2016 Summer. 16(3): pp. 141–6. PMC7191028 27840342

[30] Kiani B, et al. Spatio-temporal epidemiology of the tuberculosis incidence rate in Iran 2008 to 2018. BMC Public Health. 2021;21(1):1093. 34098917 10.1186/s12889-021-11157-1 PMC8186231

[31] Ammari L, et al. Epidemiology of tuberculosis, in Imaging of Tuberculosis. Springer; 2022. pp. 1–13.

[32] Khajedaluee M, et al. The burden of tuberculosis in Iran, A 12-year population-based study. Med J Islamic Repub Iran. 2021;35:13. 10.47176/mjiri.35.13 PMC8111624 33996664

[33] Sabermahani A, et al. Provincial human development index, a guide for efficiency level analysis: the case of Iran. Iran J public health. 2013;42(2):149. 23515434 PMC3595646

[34] Morse S. Quality of life, well-being and the human development index: a media narrative for the developed world? Soc Indic Res. 2023;170(3):1035–58.

[35] Fujita DM, Alvarenga RF, de Andrade HF Jr. Trends in tuberculosis and inequality-adjusted Human Development Index in Brazil, 2018–2022. J Public Health Policy. 2025;46(1):139–48. 39710742 10.1057/s41271-024-00543-9

[36] Rodríguez-Morales AJ, Castañeda-Hernández DM. Relationships between morbidity and mortality from tuberculosis and the human development index (HDI) in Venezuela, 1998–2008. Int J Infect Dis. 2012;16(9):e704–5. 22721701 10.1016/j.ijid.2012.04.011

[37] Fathollahi F, et al. Burden of prostate cancer and relationship with the human development index (HDI) in Asia: A study of Global Burden disease in 2019. Caspian J Intern Med. 2023;14(4):710. 38024182 10.22088/cjim.14.4.710 PMC10646363

[38] Zhang Q, et al. An ecological study of tuberculosis incidence in China, from 2002 to 2018. Front Public Health. 2022;9:766362. 35118041 10.3389/fpubh.2021.766362 PMC8804159

[39] Chen Z, et al. Age-period-cohort analysis and prediction of tuberculosis trends in China—based on the Global Burden of Disease 2021 data. Front Public Health. 2025;13:1512514. 40027502 10.3389/fpubh.2025.1512514 PMC11868063

[40] Daneshi S, et al. Prevalence and contributing factors of drug-resistant tuberculosis (DR-TB) in iran: a systematic review. BMC Infect Dis. 2025;25(1):1004. 40781658 10.1186/s12879-025-11439-8 PMC12335167

[41] Alavi SM, et al. Incidence trend analysis of tuberculosis in Khuzestan Province, southwest of Iran: 2010–2019. Global Epidemiol. 2023;6:p100118. 10.1016/j.gloepi.2023.100118 PMC10445994 37637715

[42] Rastegar M, et al. Effective Reproduction Number of Smear-Positive Pulmonary Tuberculosis in Iran: A Registry-Based Study (2011–2021). J Res Health Sci. 2024;24(4):e00633. 39431658 10.34172/jrhs.2024.168 PMC11492524

[43] Fallahi MJ, et al. Changes in incidence and clinical features of tuberculosis with regard to the COVID-19 outbreak in Southern Iran. BMC Infect Dis. 2024;24(1):1043. 39333984 10.1186/s12879-024-09947-0 PMC11430532

[44] Ayoubi S, et al. Prevalence and temporal trends of multidrug-resistant tuberculosis in Iran from 1981 to 2023: a systematic review and meta-analysis. Int J Mycobacteriology. 2024;13(3):320–30. 10.4103/ijmy.ijmy_162_24 39277896

